# Long noncoding RNA ASB16-AS1 inhibits adrenocortical carcinoma cell growth by promoting ubiquitination of RNA-binding protein HuR

**DOI:** 10.1038/s41419-020-03205-2

**Published:** 2020-11-20

**Authors:** Bo Long, Xufei Yang, Xixia Xu, Xiaoxin Li, Xinjie Xu, Xuebin Zhang, Shuyang Zhang

**Affiliations:** 1 Medical Science Research Center, Peking Union Medical College Hospital, Chinese Academy of Medical Sciences and Peking Union Medical College, Beijing, 100730 China; 2 Graduate School of Peking Union Medical College, Chinese Academy of Medical Sciences and Peking Union Medical College, Beijing, 100730 China; 3 Department of Urology Surgery, Peking Union Medical College Hospital, Peking Union Medical College and Chinese Academy of Medical Sciences, Beijing, 100730 China; 4 Department of Cardiology, Peking Union Medical College Hospital, Peking Union Medical College and Chinese Academy of Medical Sciences, Beijing, 100730 China

**Keywords:** Long non-coding RNAs, Ubiquitylation, Adrenal tumours

## Abstract

Adrenocortical carcinoma is one of the aggressive malignancies and it originates from the cortex of adrenal gland. Dysregulation of long non-coding RNA plays important roles in the development of adrenocortical carcinoma. Here, we found that lncRNA ASB16-AS1 was down-regulated in adrenocortical carcinoma and ASB16-AS1 functions as tumor suppressor in vitro and in vivo. We then found that IGF1R and CDK6 are regulated by ASB16-AS1 in adrenocortical carcinoma cells by transcriptome RNA sequencing. ASB16-AS1 associates with RNA-binding protein HuR (ELAVL1) as revealed by RNA pull-down following mass spectrometry. Also, ASB16-AS1 inhibits HuR expression post-translationally by promoting its ubiquitination. ASB16-AS1 regulates IGF1R and CDK6 mRNA expression through RNA-binding protein HuR. We then found that inhibition of ASB16-AS1 attenuates the binding of ubiquitin E3 ligase BTRC to HuR and subsequently inhibits HuR protein unbiquitination and degradation. BTRC knock-down could reverse the effect of AB16-AS1 on HuR, CDK6, and IGF1R levels. Collectively, these results demonstrate that ASB16-AS1 regulates adrenocortical carcinoma cell proliferation and tackling the level of ASB16-AS1 may be developed to treat adrenocortical carcinoma.

## Introduction

Adrenocortical carcinoma is a rare and aggressive malignancy that comes from the cortex of adrenal gland. This type of malignancy lacks effective treatment and mostly results in poor outcomes^1^. It is of great importance to elucidate the molecular mechanism driving the growth of adrenocortical carcinoma and explore potential therapeutic targets for treating this type of cancer.

Long non-coding RNAs (LncRNAs) are a class of RNAs that are more than 200 nucleotides in length and encode no protein products. LncRNAs participate in diverse cellular processes and dysregulation of lncRNAs results in the pathogenesis of many diseases including cancer^2–4^. LncRNAs regulate cell proliferation and functions as tumor suppressors or oncogenes in cancers. LncRNAs exert their function in *cis* regulating nearby gene expression or leaving the site of transcription and perform cellular function in *trans*^5–7^. Recent studies have found that lncRNAs mediate cancer signaling pathways by interaction with proteins. These proteins underwent post-translational modifications and the abundance of proteins are modulated by lncRNAs^8,9^. Human antigen R (HuR) is the ubiquitous member of embryonic lethal abnormal vision (ELAV) family of RNA-binding proteins. HuR binds transcripts in the AU-rich element and promotes the stability of target mRNAs^10,11^. It associates with specific mRNAs encoding proteins that promote cancer cell proliferation and cell survival^12,13^. The protein level of HuR can be post-translationally regulated by ubiquitin–proteasome system^10,14^. Ubiquitination is sequentially performed by E1 ubiquitin-activating enzymes, E2 ubiquitin-conjugating enzymes, and E3 ubiquitin ligases^15^. Studies found that E3 ligase β-TrCP1 (BTRC) target HuR for ubiquitin-mediated protein degradation^14,16^. Several studies have found that ASB16-AS1 regulate proliferation in glioma, hepatocellular carcinoma, cervical cancer, non-small cell lung cancer, and in osteosarcoma^17–21^. However, whether ASB16-AS1 plays an important role in adrenocortical carcinoma remains to be clarified.

In this study, we found that ASB16-AS1 was down-regulated in adrenocortical carcinoma, and inhibition of the expression of ASB16-AS1 promotes cell proliferation in vitro. In addition, overexpression of ASB16-AS1 inhibits tumor growth in vivo as revealed by xenograft tumor experiment. We then found that IGF1R and CDK6 were up-regulated upon knockdown of ASB16-AS1 in adrenocortical carcinoma cells. ASB16-AS1 associates with HuR protein and ASB16-AS1 regulates the expression of IGF1R and CDK6 through HuR. ASB16-AS1 post-translationally regulates HuR protein levels by modulating the association of ubiquitin E3 ligase BTRC with HuR. Our results may be developed to treat adrenocortical carcinoma.

## Materials and methods

### Adrenocortical carcinoma specimens

Two cohorts of adrenocortical carcinoma samples were collected in this study. Cohort 1 contains fresh adrenocortical carcinoma tissues from 21 patients. The normal adrenal glands were collected from patients who were diagnosed as renal carcinoma and underwent nephrectomy. Cohort 2 contains paraffin-embedded tissue samples from 57 patients. All the patients included in this study received no radiotherapy or chemotherapy before the operation. Written informed consent to the use of the tissue samples for research purposes was obtained from all patients. The study protocol was approved by the Ethics Committee of Peking Union Medical College Hospital.

### Cell culture

Adrenocortical carcinoma cell line SW-13 and H295R cells were obtained from China Infrastructure of Cell Line Resource. SW-13 was cultured in Leibovitz’s L-15 medium (Gibco) supplemented with 10% fetal bovine serum (Gibco) and H295R were cultured in DMEM/F12 Medium (Gibco) supplemented with 2.5% Nu-Serum I (Corning) and ITS+ premix (Corning). Cells were cultured at 37 °C in a humidified atmosphere containing 5% CO_2_.

### Quantitative real-time PCR (qRT-PCR)

Total RNA was extracted using TRIzol (Invitrogen). After Dnase I treatment (Takara, Japan), the RNA was reverse-transcribed using a reverse transcriptase (ReverTra Ace, Toyobo). The experiments were run in triplicate using an Applied Biosystems ABI 7500 sequence detector system according to the manufacturer’s instructions. The results of qRT-PCR were normalized to those of GAPDH. The specificity of the PCR amplification was confirmed by agarose gel electrophoresis. Primers used in this study are listed in Supplementary Table 3.

### Immunoblot

Adrenocortical carcinoma cells were lysed at 4 °C in a lysis buffer (20 mM Tris, pH 7.5, 2 mM EDTA, 3 mM EGTA, 2 mM dithiothreitol, 250 mM sucrose, 0.1 mM phenylmethylsulfonyl fluoride, 1% Triton X-100, and a protease inhibitor mixture). The samples were subjected to 12% SDS–PAGE and transferred to nitrocellulose membranes. Equal protein loading was controlled using Ponceau red staining of the membranes. Anti-HuR (3A2) was from santa cruz (sc-5261), anti-BTRC was from thermofisher (37-3400), anti-CDK6 was from abcam (ab124821), and anti-IGF1R was from abcam (ab182408).

### CCK-8 assay

Cell proliferation was performed using the CCK-8 assay kit (Dojindo, Tokyo, Japan) according to the manufacturer’s instructions. SW-13 or H295R cells were seeded in a 96-well plate. CCK-8 reagent was then added into the culture medium for two hours. Absorbance was measured at 450 nm using a Varioskan^®^ Flash Spectral Reader (Thermo Scientific, USA) after transfection at the indicated time point.

### 5-ethylnyl-2ʹ-deoxyuridine (EdU) incorporation assay

EdU assay was performed by Click-iT^®^ Plus EdU Imaging Kits. Briefly, EdU was added into the culture medium at the concentration of 10 μM and two hours later EdU was detected according to the manufacturer’s instructions. The nuclei were stained by Hoechst^®^ 33342 at room temperature for 30 minutes.

### Nucleus–cytoplasm fractionation

Cytoplasmic and nucleic RNAs were extracted from SW-13 or H295R cells using PARIS™ Kit (Ambion) according to the manufacturer’s instruction. After washed with prechilled PBS, the cells were fractionated by centrifugation to obtain the supernatant and nuclear pellet and RNA was extracted, respectively. The supernatant was extracted as cytoplasmic RNA and nuclear pellet was extracted as nuclear RNA.

### RNA stability assays

Adrenocortical carcinoma cells were treated with actinomycin D at the concentration of 5 μg/ml. The cells were harvested at the indicated time points and RNA was extracted by TRIzol reagent. The mRNA levels were detected by qRT-PCR.

### Cell transfection

SiRNAs targeting ASB16-AS1, HuR, and BTRC were synthesized by Genepharma (Shanghai, China). The siRNAs were transfected at the concentration of 50 nM using Lipofecamine RNAiMAX transfection reagent according to the manufacturer’s instruction. For overexpression of ASB16-AS1, the coding sequence of ASB16-AS1 was ligated into pcDNA3.1 vector. Empty pcDNA3.1 vector was served as negative control (NC). The plasmid transfection was performed by Lipofectamine 3000 transfection reagent. The siRNA sequences are listed in Supplementary Table 4.

### Cell cycle analysis

Adrenocortical carcinoma cells were fixed in 70% ethanol at 4 °C overnight and washed with PBS three times. The cells were then treated with FxCycle™ PI/RNase staining solution (Thermo Scientific) for 30 min. The cell cycle was analyzed using an Accuri C6 cytometer (BD Biosciences).

### In situ hybridization

The expression of ASB16-AS1 was detected and analyzed by RNAscope^®^ 2.5 HD Detection Reagent (Advanced Cell Diagnostics) according to the manufacturer’s instruction and as described^22^. The RNAscope probe targeting ASB16-AS1 was designed and synthesized by Advanced Cell Diagnostics company (cat. no. 888271). The paraffin-embedded tissues were cut into 4 μm sections and baked for 1 h at 60 °C. Then the sections were deparaffinized with xylene and dehydrated in ethanol. After treatment with hydrogen peroxide for 10 min at room temperature, target retrieval was performed by putting the sides into the boiling Target Retrieval solution for 15 min. The slides were then washed in distilled water and ethanol. Protease Plus was added to each section for 30 min at 40 °C. After washing with distilled water, the slides were incubated with probe targeting ASB16-AS1 for 2 h at 40 °C in the HybEZ oven. The sides were then hybridized with Amp 1 to Amp 6. After that, the tissue sections were incubated with DAB. Then the sides were stained with 50% hematoxylin for 2 min at room temperature and washed with 0.02% ammonia water and distilled water. The slides were then mounted with Cytoseal and examined under a standard bright field microscope. ASB16-AS1 expression was semi-quantified according to the manufacturer’s recommendation. Score 0: No staining or <1 dot to every 10 cells. Score 1: 1–3 dots/cell. Score 2: 4–10 dots/cell. Score 3: >10 dots/cell and <10% positive cells have dot clusters. Score 4: >10 dots/cell and more than 10% positive cells have dot clusters. Scores of 0 and 1 were classified into the low expression and scores of 2–4 were defined as high expression. Two separate individuals who were blinded to the slides scored the samples.

### RNA sequencing and data analysis

Total RNA was extracted from NC or ASB16-AS1 siRNA transfected SW-13 cells. Sequencing libraries were generated using the NEBNext^®^ Ultra^TM^ RNA Library Prep Kit for Illumina^®^ (NEB, USA). The libraries were sequenced on an Illumina Hiseq platform. Differential expression analysis was performed using the DESeq2 R package. Gene ontology (GO) analysis of differentially expressed genes was conducted using the clusterProfiler R package. GO terms with corrected *P* values < 0.05 were considered significantly enriched by differentially expressed genes.

### RNA pull-down and mass spectrometry analysis

RNA pull-down was performed as described elsewhere^23^. Briefly, ASB16-AS1 and its antisense RNA were biotinylated by using MEGAscript™ T7/SP6 Transcription Kit (Life Technologies, USA) according to the manufacturer’s instruction. The biotinylated RNAs were then incubated with cell lysate at 4 °C for two hours. Proteins that interact with ASB16-AS1 were precipitated by Dynabeads™ M-280 Streptavidin beads (Life Technologies, USA) by incubating at 4 °C for one hour. The pull-down products were then subjected to SDS–PAGE and gel lanes were cut to pieces for mass spectrometry analysis to identify proteins specifically bind with ASB16-AS1.

### Immunohistochemistry (IHC) analysis

The xenografted tumors were fixed in 4% paraformaldehyde and then embedded in paraffin. The sections were then routinely deparrafinized by incubating with xylene. Antigen retrieval was performed by incubating the sections in citrate buffer. Hydrogen peroxide was used to suppress endogenous peroxidase. The sections were then treated with normal goat serum in TBS buffer for 1 h at room temperature to prevent nonspecific antibody binding. The tumor sections were then incubated with Ki-67 antibody, CDK6, or IGF1R antibody, respectively, at 4 °C. After washing with PBS, the sections were incubated with secondary antibody following DAB treatment.

### RNA immunoprecipitation

RNA immunoprecipitation assays were performed by Magna RIP RNA-Binding Protein Immunoprecipitation Kit (Millipore, USA) according to the manufacturer’s instructions. In brief, cells were harvested in RIP lysis buffer and were incubated with HuR antibody or IgG overnight at 4 °C. Input RNA and immunoprecipitated RNA were detected by qRT-PCR using specific primers for ASB16-AS1.

### Xenografted tumor model

Four weeks old BALB/c female athymic nude mice (Vital River Laboratories) were housed in specific pathogen-free conditions. Mice were randomly divided into two groups with six mice for each group. Adrenocortical carcinoma cells stably expressing ASB16-AS1 were injected subcutaneously into the flank region of the mice. Tumor volumes were calculated as length × width^2^ × 0.5 in mice. Tumor volumes were detected blindly. All animal studies were approved by Animal Care and Use Committee of Peking Union Medical College Hospital.

### Statistical analysis

Data are expressed as mean ± SEM of at least three independent experiments. Two-tailed *χ*^2^ test or Fisher’s exact test was used to define the relationship between clinicopathological characteristics and ASB16-AS1 expression level. Survival analysis was performed by the Kaplan–Meier method and a log-rank test was used to determine the significance of the differences in survival. Student’s *t*-test was used for two group comparisons. The statistical comparison among different groups was performed using one-way ANOVA. The data met the assumptions of the tests. The variance was similar between the groups that are being statistically compared. *P* < 0.05 was considered statistically significant.

## Results

### LncRNA ASB16-AS1 is downregulated in adrenocortical carcinoma and associates with prognosis in adrenocortical carcinoma patients

**Fig. 1:**
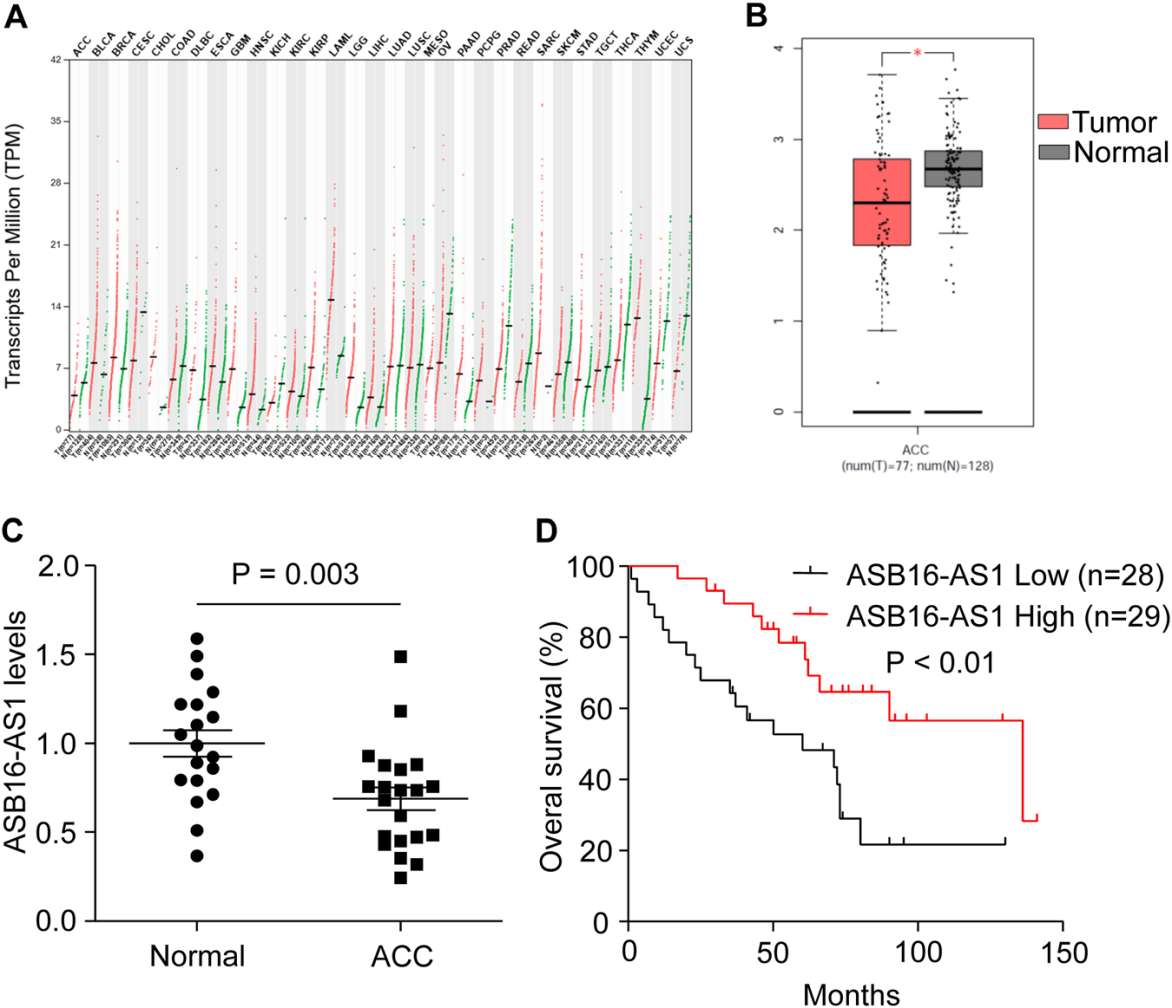
LncRNA ASB16-AS1 is down-regulated in adrenocortical carcinoma and correlates with survival time of patients. **a** The expression of ASB16-AS1 in different types of cancers. The data were obtained from The Cancer Genome Atlas (TCGA) data portal in GEPIA. The expression of ASB16-AS1 in cancer is shown with red dots and in normal tissue is shown with green dots. The full names of different tumors are listed in Supplementary Table 6. **b** The expression of ASB16-AS1 is down-regulated in adrenocortical carcinoma compared with normal adrenal tissue with |Log_2_FC | Cutoff: 0.37 and *P*-value cutoff: 0.01. **c** The expression of ASB16-AS1 was down-regulated in adrenocortical carcinoma compared with normal adrenal glands in surgically obtained samples. The relative expression of ASB16-AS1 was detected by qRT-PCR in adrenocortical carcinoma (*n* = 21) and normal adrenal glands (*n* = 19). **d** Kaplan–Meier analysis of adrenocortical carcinoma patients with low (*n* = 28) or high (*n* = 29) ASB16-AS1 levels. Statistical analysis was performed by log-rank test.

To investigate whether ASB16-AS1 participates in the pathogenesis of adrenocortical carcinoma, we analyzed the expression of ASB16-AS1 in adrenocortical carcinoma using gene expression profiling interactive analysis (GEPIA) tool whose data were obtained from TCGA and GTEx. The result turned out that the expression of ASB16-AS1 is different in various kinds of tumors (Fig. 1a). However, the expression of ASB16-AS1 is down-regulated in adrenocortical carcinoma compared with normal adrenal glands as revealed by GEPIA (Fig. 1b). To verify the result, we examined the expression level of ASB16-AS1 in surgically obtained samples by qRT-PCR. The results showed that the expression of ASB16-AS1 is significantly down-regulated in adrenocortical carcinoma samples compared with normal adrenal glands (Fig. 1c).

To clarify the relationship between ASB16-AS1 expression and overall survival time and clinicopathological characters, RNAscope in situ hybridization was performed to detect the expression of ASB16-AS1 in 57 adrenocortical carcinoma specimens (cohort 2). Using the Kaplan–Meier survival analysis, we found that patients with lower ASB16-AS1 expression had shorter overall survival time compared with patients with higher ASB16-AS1 expression (Fig. 1d). We then examined the clinicopathological characteristics of ASB16-AS1 in adrenocortical carcinoma patients. ASB16-AS1 expression was negatively correlated with tumor size, European Network for the Study of Adrenal Tumors (ENSAT) tumor stage, Ki-67 index, lymph node metastasis, and distant metastasis (Table 1).

**Table 1:** Relationship between ASB16-AS1 and clinicopathological features of adrenocortical carcinoma patients.

Characteristics	No. of cases	ASB16-AS1 expression		*P* value
		Low (*n* = 28)	High (*n* = 29)	
*Age*				0.689
≥50	29	15	14	
<50	28	13	15	
*Gender*				0.881
Male	25	12	13	
Female	32	16	16	
*Tumor size*				0.012*
≥7.5 cm	29	19	10	
<7.5 cm	28	9	19	
*ENSAT tumor stage*				0.005*
I + II	35	12	23	
III + IV	22	16	6	
*Laterality*				0.872
Left	23	11	12	
Right	34	17	17	
*Ki-67*				0.012*
≥20%	27	18	9	
<20%	30	10	20	
*Lymph node metastasis*				0.041*
Positive	10	8	2	
Negative	47	20	27	
*Distant metastasis*				0.01*
Yes	12	10	2	
No	45	18	27	

*ENSAT* European Network for the Study of Adrenal Tumors.

*P < 0.05 was considered significant.

### LncRNA ASB16-AS1 regulates the proliferation of adrenocortical carcinoma cells in vitro

**Fig. 2:**
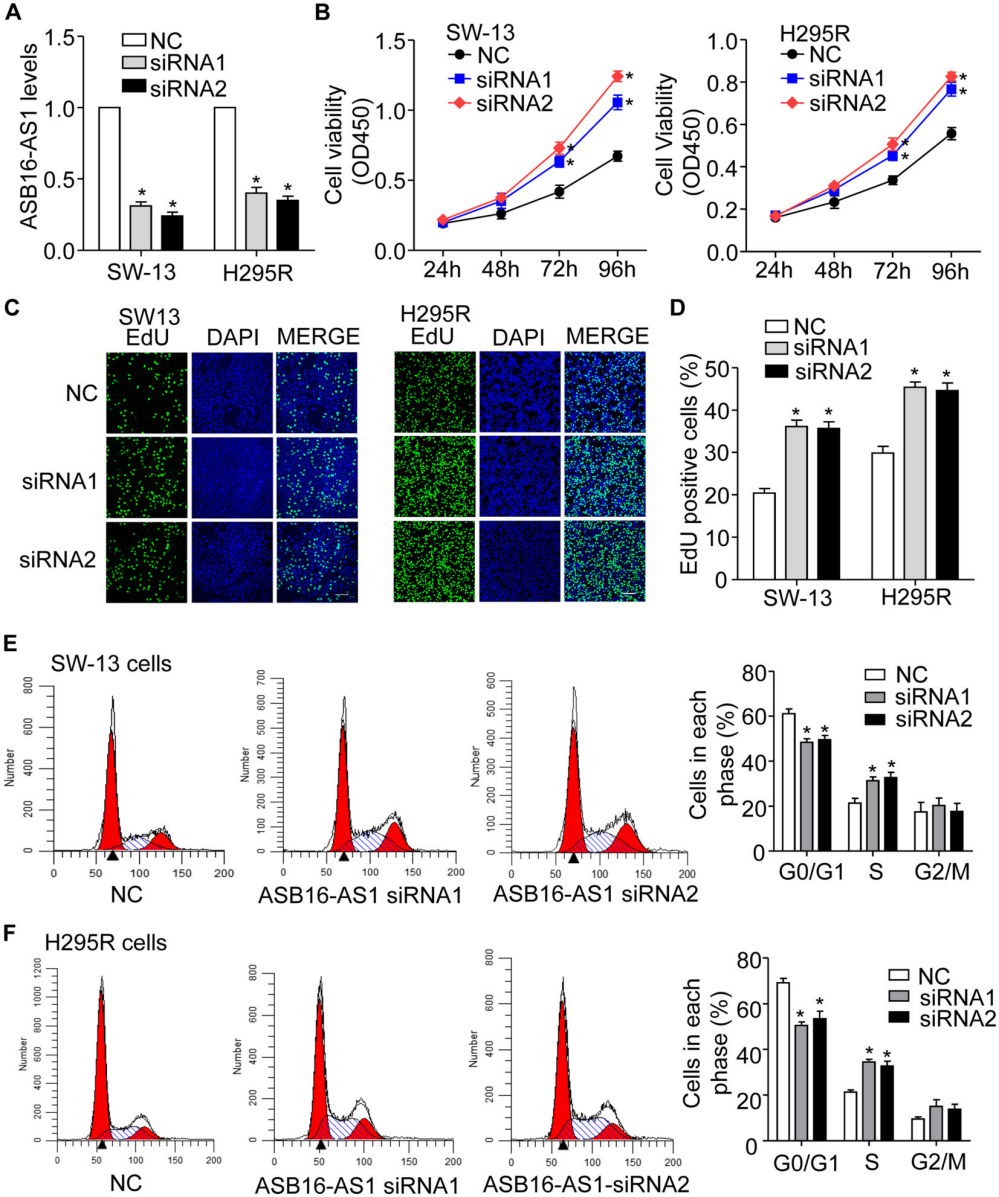
Inhibition of ASB16-AS1 expression promotes adrenocortical carcinoma cell proliferation. **a** siRNAs specifically inhibits the expression of ASB16-AS1 in adrenocortical carcinoma cells. 50 nM of ASB16-AS1 siRNA1 (siRNA1) or ASB16-AS1 siRNA2 (siRNA2) were transfected into adrenocortical carcinoma cell SW-13 or H295R, 48 h post transfection the expression of ASB16-AS1 were analyzed by qRT-PCR. *P < 0.05 versus NC group. **b** Inhibition of ASB16-AS1 expression promoted the proliferation of adrenocortical carcinoma cells analyzed by CCK-8 assay. Adrenocortical carcinoma cells were transfected with ASB16-AS1 siRNA1 (siRNA1) or ASB16-AS1 siRNA2 (siRNA2) at the concentration of 50 nM, cell proliferation was analyzed by CCK-8 assay at the indicated time post-transfection. *P < 0.05 versus NC group. **c**, **d** Inhibition of ASB16-AS1 expression promotes the proliferation of adrenocortical carcinoma cells analyzed by EdU incorporation assay. Adrenocortical carcinoma cells were transfected with ASB16-AS1 siRNA1 (siRNA1) or ASB16-AS1 siRNA2 (siRNA2) at the concentration of 50 nM, cell proliferation was analyzed by EdU assay. Scale bar equals 100 μm. *P < 0.05 versus NC group. **e**, **f** Inhibition of ASB16-AS1 promotes cell cycle progression in adrenocortical carcinoma cells. Adrenocortical carcinoma cells were transfected with 50 nM of ASB16-AS1 siRNA1 (siRNA1) or ASB16-AS1 siRNA2 (siRNA2), cell cycle was analyzed by FACS assay. *P < 0.05 versus NC group. The experiments were performed in triplicate. The data are represented as mean ± SEM from three independent experiments.

To explore whether dysregulation of ASB16-AS1 participate in the proliferation of adrenocortical carcinoma cells, siRNAs specifically targeting endogenous ASB16-AS1 were transfected into SW-13 and H295R cells, respectively (Fig. 2a). The results demonstrate that inhibition of endogenous ASB16-AS1 significantly promotes adrenocortical carcinoma cell proliferation in vitro as determined by CCK-8 and EdU incorporation assay (Fig. 2b–d). To further confirm the role of ASB16-AS1 in controlling adrenocortical carcinoma cell proliferation and cell cycle progression, we knocked down the expression of ASB16-AS1 and analyzed cell cycle distribution by flow cytometry in adrenocortical carcinoma cells. The results showed that inhibition of endogenous ASB16-AS1 promoted the percentage of cells in S phase and a reduction in the G0/G1 cell phase (Fig. 2e, f). In summary, these data indicate that inhibition of ASB16-AS1 promotes cell proliferation and cell cycle progression in adrenocortical carcinoma cells in vitro.

To further confirm the role of ASB16-AS1 in the regulation of adrenocortical carcinoma cell proliferation and cell cycle progression, we constructed a plasmid expressing ASB16-AS1 (Supplementary Fig. S1a). We transfected the plasmid into adrenocortical carcinoma cell SW-13 and H295R and the results turned out that enhanced expression of ASB16-AS1 inhibited adrenocortical carcinoma cell proliferation as revealed by CCK-8 and EdU assays (Supplementary Fig. S1b, c). In addition, cell cycle progression is also significantly repressed with enhanced expression of ASB16-AS1 in adrenocortical carcinoma cells (Supplementary Fig. S1d, e). All these data demonstrate that ASB16-AS1 regulates adrenocortical carcinoma cells proliferation and cell cycle progression in vitro.

### ASB16-AS1 inhibits adrenocortical carcinoma tumor growth in vivo

**Fig. 3:**
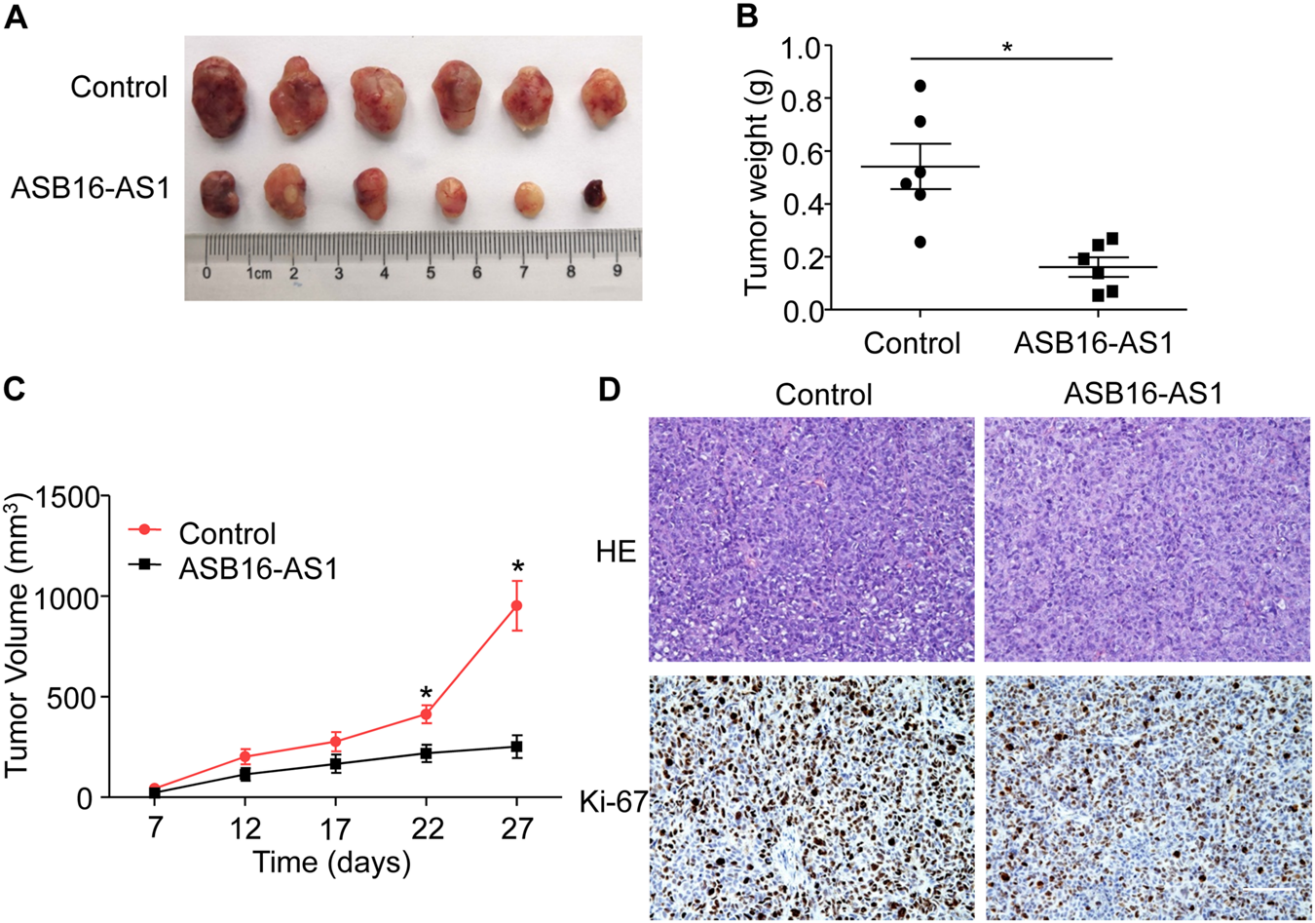
ASB16-AS1 inhibits xenograft tumor growth. **a** SW-13 cells stably expressing ASB16-AS1 or control cells were injected into nude mice and xenograft tumors were dissected from nude mice 27 days after injection (*n* = 6 per group). **b**, **c** Enhanced ASB16-AS1 reduces the tumor weight and tumor volumes in xenograft tumors. SW-13 cells stably expressing ASB16-AS1 was injected into nude mice and tumor weight was measured 27 days post injection (**b**) and tumor volumes was measured 7 days after injection (**c**). *P < 0.05 versus control. **d** Histopathology of xenograft tumors derived from SW-13 cells stably expressing ASB16-AS1. Xenograft tumors derived from SW-13 cells were sectioned and stained by HE staining and immunohistochemical staining using Ki-67 staining. Scale bar equals 100 μm.

To study whether ASB16-AS1 participate in tumorigenesis in vivo, we constructed a SW-13 cell line stably expressing ASB16-AS1 (Supplementary Fig. S2c). These cells were harvested and subcutaneously injected into immunocompromised nude mice. The results showed that tumors generated from ASB16-AS1 cells grow significantly slower than the control counterpart with reduced tumor volume and tumor weight (Fig. 3a–c). IHC results showed that overexpression of ASB16-AS1 reduced the percentage of Ki-67 positive cells (Fig. 3d). All of these data indicates that ASB16-AS1 inhibits tumor growth in vivo.

### ASB16-AS1 regulates the expression of genes participating in cell cycle progression and cell proliferation

**Fig. 4:**
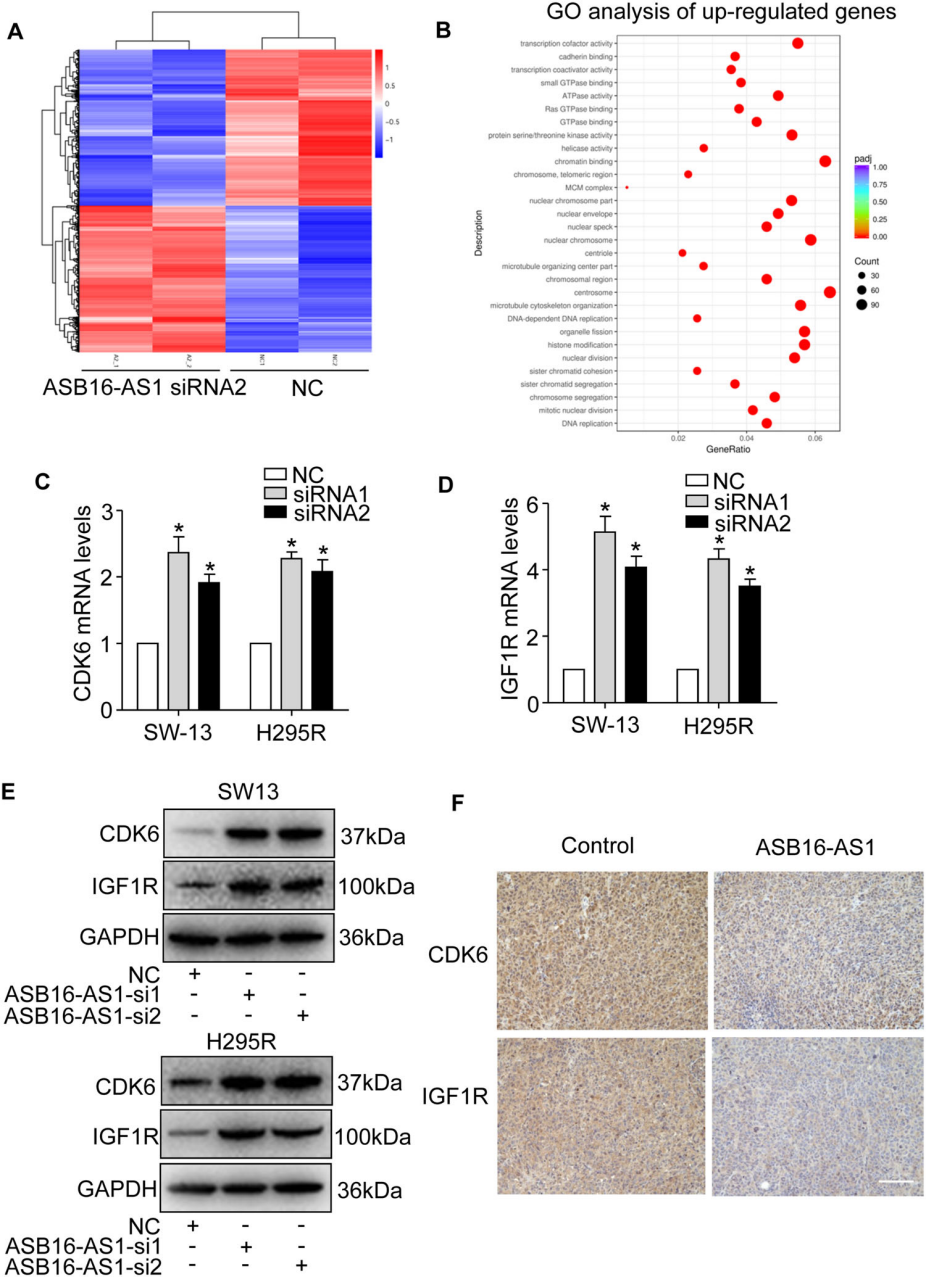
ASB16-AS1 regulates CDK6 and IGF1R mRNAs expression in adrenocortical carcinoma cells. **a** Heatmap of RNA transcriptome sequencing upon inhibition of ASB16-AS1 expression. SW-13 adrenocortical carcinoma cells were transfected with ASB16-AS1 siRNA2 and RNA sequencing was performed 48 h post-transfection. **b** Gene ontology (GO) analysis of up-regulated genes when the expression of ASB16-AS1 is inhibited. **c**, **d** Inhibition of ASB16-AS1 up-regulates CDK6 and IGF1R mRNAs expression. ASB16-AS1 siRNA1 (siRNA1) or ASB16-AS1 siRNA2 (siRNA2) was transfected into adrenocortical carcinoma cell SW-13 or H295R cells and qRT-PCR was performed to analyze the expression of CDK6 and IGF1R expressions 48 h post transfection. *P < 0.05 versus NC group. **e** Inhibition of ASB16-AS1 up-regulates the protein levels of CDK6 and IGF1R. ASB16-AS1 siRNA1 (si1) or ASB16-AS1 siRNA2 (si2) was transfected into SW-13 or H295R cells, the protein levels of CDK6 and IGF1R were analyzed by western blot. **f** Immunohistochemical staining of CDK6 and IGF1R in sections from xenograft tumors stably expressing ASB16-AS1. Scale bar equals 100 μm. The experiments were performed in triplicate. The data are represented as mean ± SEM from three independent experiments.

To elucidate the mechanism ASB16-AS1 controlling adrenocortical carcinoma cell proliferation and tumor growth, we performed RNA sequencing to profile the transcriptome changes when ASB16-AS1 was knocked down in adrenocortical carcinoma cells. We selected ASB16-AS1 siRNA2 whose knockdown efficiency is relatively higher than ASB16-AS1 siRNA1. The results showed that ASB16-AS1 knockdown initiates considerable gene expression changes, with 2167 genes down-regulated and 2028 genes up-regulated (Fig. 4a and Supplementary Table 1). By GO analysis, these up-regulated genes were enriched in cell cycle control and cell proliferation (Fig. 4b). Among these genes, we found that IGF1R and CDK6 that are involved in regulation of cancer cell proliferation and tumor growth^24–26^. We validated the RNA-sequencing results by qRT-PCR and the results demonstrate that IGF1R and CDK6 mRNA levels were increased upon knockdown of ASB16-AS1 (Fig. 4c, d). In addition, western blot showed that IGF1R and CDK6 protein levels were also increased (Fig. 4e). To further validate the regulation of IGF1R and CDK6 by ASB16-AS1, we found that enhanced expression of ASB16-AS1 inhibited the mRNA and protein levels of IGF1R and CDK6 (Supplementary Fig. S3a–d). In addition, immunochemistry results showed that over-expression of ASB16-AS1 reduced the levels of CDK6 and IGF1R in xenografted tumor tissues (Fig. 4f). Collectively, these data indicate that ASB16-AS1 regulates expression of IGF1R and CDK6 in adrenocortical carcinoma cells.

### ASB16-AS1 interacts with RNA-binding protein HuR

**Fig. 5:**
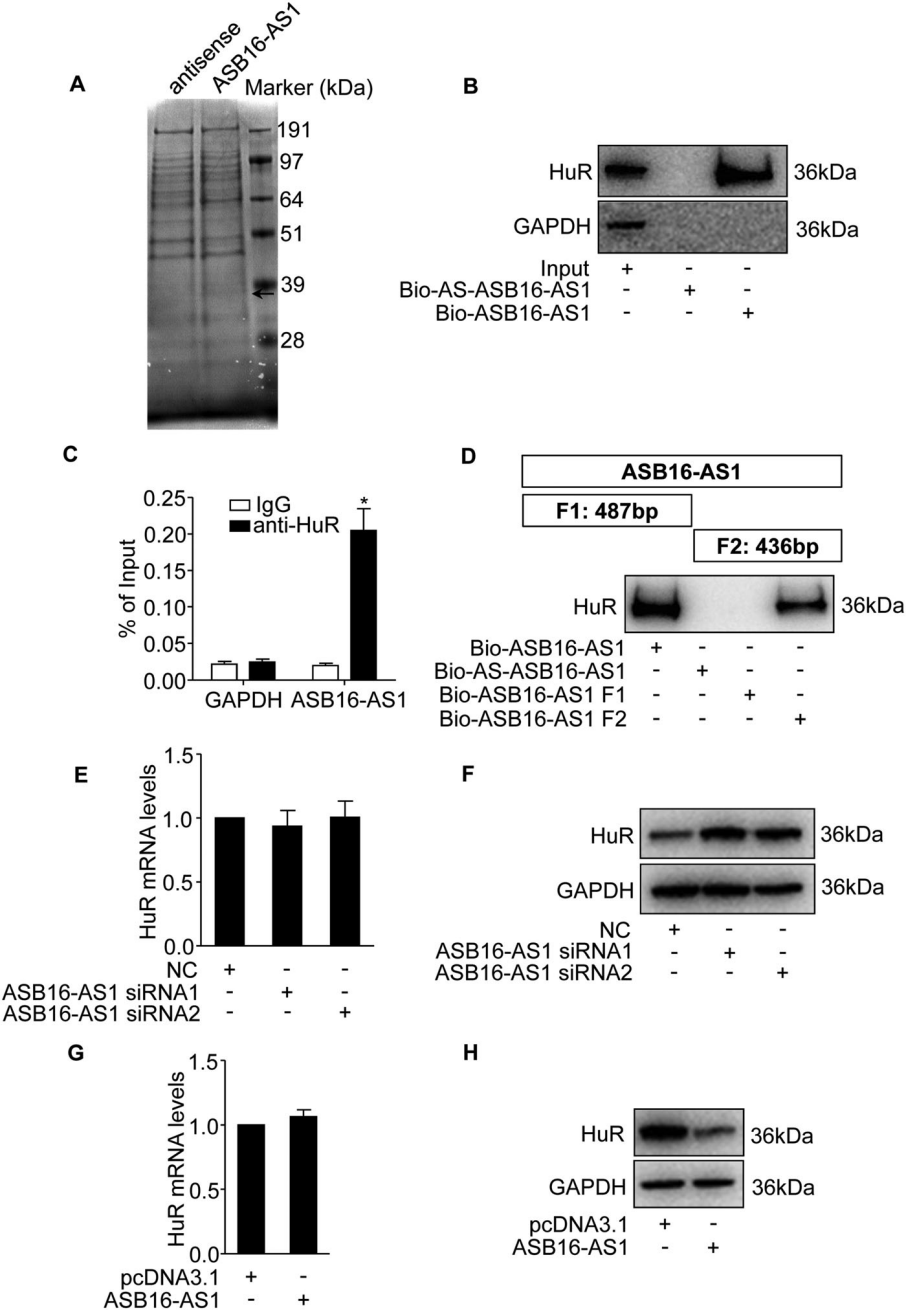
ASB16-AS1 associates with RNA-binding protein HuR and regulates protein levels of HuR. **a** Identification of proteins that associate with ASB16-AS1. Biotinylated ASB16-AS1 or biotinylated antisense ASB16-AS1 was incubated with SW-13 cell extracts and RNA pull-down was performed to identify the proteins that associate with ASB16-AS1. The lanes were excised and analyzed by mass spectrometry. The arrow indicates the potential protein that specifically associates with ASB16-AS1. **b** HuR protein binds with ASB16-AS1. Biotinylated ASB16-AS1 or its antisense sequence was incubated with SW-13 cell extracts and RNA pull-down following western blot was performed to detect the association of HuR with ASB16-AS1. GAPDH served as negative control. **c** RIP assays to verify the association between ASB16-AS1 and HuR protein. HuR antibody was incubated with SW-13 cell extracts and qRT-PCR was performed to test the relative amount of ASB16-AS1 associates with HuR protein. IgG was served as negative control. *P < 0.05 versus IgG group. **d** HuR protein binds with fragment 2 of ASB16-AS1. Biotinylated full length ASB16-AS1 or truncated ASB16-AS1 fragment was incubated with cell extracts from SW-13 cells and RNA pull-down following western blot was performed using HuR antibody. Biotinylated antisense ASB16-AS1 was used as control. **e**, **f** Knockdown of ASB16-AS1 elevated HuR protein levels. ASB16-AS1 siRNAs were transfected into SW-13 cells, HuR mRNA levels was tested by qRT-PCR (**e**) and protein levels were tested by western blot (**f**). **g**, **h** Enhanced expression of ASB16-AS1 reduces the expression of HuR protein. ASB16-AS1 overexpression plasmid was transfected into SW-13 cells, HuR mRNA and protein levels were tested by qRT-PCR and western blot, respectively. pcDNA3.1 served as control. The experiments were performed in triplicate. The data are represented as mean ± SEM from three independent experiments.

Recently, studies found that lncRNA can exert their function by interacting with proteins to regulate target gene expression^14,27^. We fractionated adrenocortical carcinoma cell cytoplasm and nucleus, and found that ASB16-AS1 is distributed both in the cytoplasm and nucleus abundantly (Supplementary Fig. S2a, b). To elucidate the mechanism ASB16-AS1 regulating IGF1R and CDK6 expressions, we performed RNA pull-down experiment following mass spectrometry to identify the proteins that associates with ASB16-AS1 (Fig. 5a and Supplementary Table 2). From these proteins, we found that ELAVL1 (HuR) is abundantly enriched in biotinylated ASB16-AS1 precipitates compared with biotinylated antisense ASB16-AS1 precipitates (Supplementary Table 2). We thus postulate that HuR protein potentially associates with ASB16-AS1. We then performed RNA pull-down assay using biotinylated ASB16-AS1 and biotinylated antisense transcript serving as control to test whether HuR could specifically bind with ASB16-AS1 as revealed by mass spectrometry. The results demonstrate that HuR exists in ASB16-AS1 captured precipitates rather than antisense ASB16-AS1 counterparts as revealed by immunobloting (Fig. 5b). To verify the interaction between ASB16-AS1 and HuR, we performed RNA immunoprecipitation to test whether endogenous ASB16-AS1 could bind HuR protein in adrenocortical carcinoma cells. The results turned out that ASB16-AS1 was significantly enriched in HuR antibody captured precipitates compared with IgG control (Fig. 5c). To characterize which region of ASB16-AS1 interacts with HuR, the full length of ASB16-AS1 was divided into two fragments according to potential HuR-binding sites (Supplementary Table 5). The biotinylated two RNA fragments were then incubated with adrenocortical carcinoma cell extracts, respectively. The proteins that potentially interact with ASB16-AS1 fragments were then pulled-down by streptavidin-linked magnetic beads. The results demonstrate that HuR interacts with fragment 2 of ASB16-AS1 rather than fragment 1 (Fig. 5d). In summary, these data demonstrate that ASB16-AS1 binds with HuR protein in adrenocortical carcinoma cells.

We found that ASB16-AS1 interacts with HuR in adrenocortical carcinoma cells, we then wonder whether ASB16-AS1 regulate HuR protein expression. Studies found that lncRNA can interact with HuR and post-translationally regulate HuR protein expression^10,14^. Our results showed that knockdown of ASB16-AS1 had no effect on HuR mRNA expression which is consistent with our RNA-sequencing results. However, HuR protein levels are significantly elevated upon knockdown of ASB16-AS1 (Fig. 5e, f). Further, we found that enhanced expression of ASB16-AS1 reduced the expression of HuR protein, whereas the mRNA level of HuR remains to be unchanged (Fig. 5g, h). These results demonstrate that ASB16-AS1 associates with HuR and regulates the expression of HuR protein expression post-translationally.

### ASB16-AS1 regulates mRNA levels of CDK6 and IGF1R through HuR

**Fig. 6:**
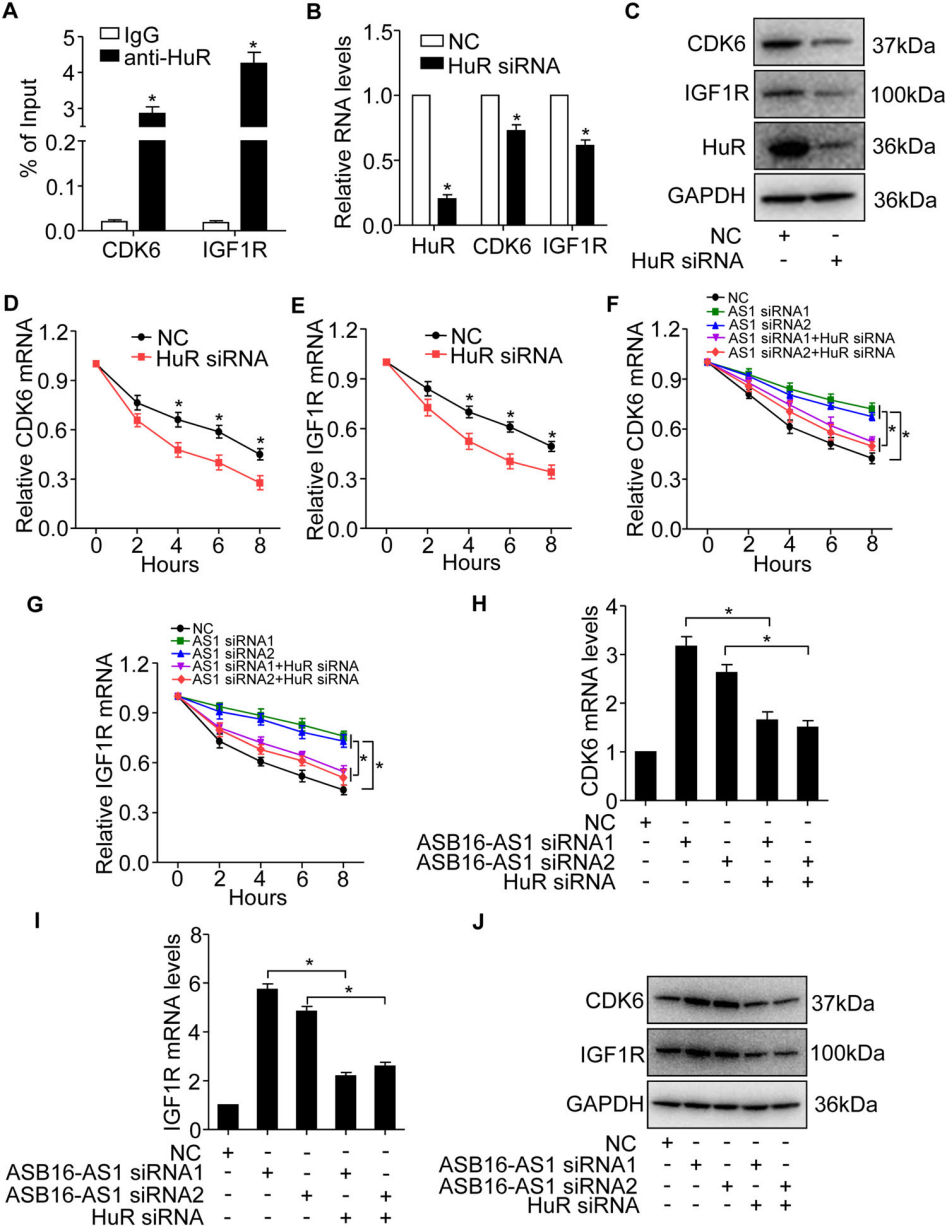
HuR regulates the expression of CDK6 and IGF1R mRNAs expression. **a** HuR RNA-binding protein associates with CDK6 and IGF1R mRNAs. RIP assays were performed in SW-13 cells using HuR antibody to detect the association between HuR protein and CDK6 or IGF1R. IgG served as negative control. *P < 0.05 versus IgG group. **b**, **c** Knockdown of HuR expression reduces CDK6 and IGF1R mRNA and protein levels. HuR siRNA was transfected into SW-13 cells at the concentration of 50 nM and CDK6 and IGF1R mRNAs expression were detected by qRT-PCR (**b**), protein levels were detected by western blot (**c**). *P < 0.05 versus NC group. **d**, **e** Inhibition of HuR reduced the mRNA stability of CDK6 and IGF1R. HuR siRNA was transfected into adrenocortical carcinoma SW-13 cells, actinomycin D was used to treat cells at different time points 48 h post transfection. RNA expression was detected by qRT-PCR. *P < 0.05 versus NC group. **f**, **g** The effect of ASB16-AS1 and HuR on CDK6 and IGF1R mRNAs stability. SW-13 cells were transfected with ASB16-AS1 siRNA1 (AS1 siRNA1) or ASB16-AS1 siRNA2 (AS1 siRNA2), respectively, or simultaneously transfected with HuR siRNA, actinomycin D was then used to treat cells at different time points 48 h post-transfection. RNA was detected by qRT-PCR. *P < 0.05. **h**–**j** Inhibition of HuR attenuates ASB16-AS1 knockdown induced CDK6 and IGF1R up-regulation. ASB16-AS1 siRNA alone or with HuR siRNA were transfected into SW-13 cells, the expression of CDK6 and IGF1R mRNAs were analyzed by qRT-PCR (**h** and **i**), protein levels were detected by western blot (**j**). *P < 0.05. The experiments were performed in triplicate. The data are represented as mean ± SEM from three independent experiments.

It is well known that HuR preferentially binds with AU-rich mRNA and stabilize target mRNAs. To figure out the mechanism ASB16-AS1 regulates CDK6 and IGF1R expressions, we performed RIP assay and found that HuR interacts with mRNAs of CDK6 and IGF1R (Fig. 6a). Knockdown of HuR down-regulates the mRNA and protein levels of CDK6 and IGF1R (Fig. 6b, c). Since HuR is a well-known RNA-binding protein that stabilizes its target mRNAs, we tested whether HuR is able to regulate the stability of CDK6 and IGF1R mRNAs. We knocked down the expression of HuR in adrenocortical carcinoma cells and the results showed that inhibition of HuR reduced the stability of CDK6 and IGF1R mRNAs (Fig. 6d, e). These data demonstrates that HuR binds and stabilize the mRNAs of CDK6 and IGF1R.

We then explored the effect of ASB16-AS1 and HuR on CDK6 and IGF1R mRNAs stability. The results showed that inhibition of ASB16-AS1 enhanced CDK6 and IGF1R mRNAs stabilities, and this effect is abolished upon inhibition of HuR (Fig. 6f, g). In addition, we found that knockdown of HuR abolished ASB16-AS1 knockdown induced up-regulation of CDK6 and IGF1R mRNA and protein levels (Fig. 6h–j). These data indicate that ASB16-AS1 regulates CDK6 and IGF1R expression through HuR which stabilizes CDK6 and IGF1R mRNAs.

### ASB16-AS1 promotes degradation of HuR by recruiting ubiquitin E3 ligase BTRC

**Fig. 7:**
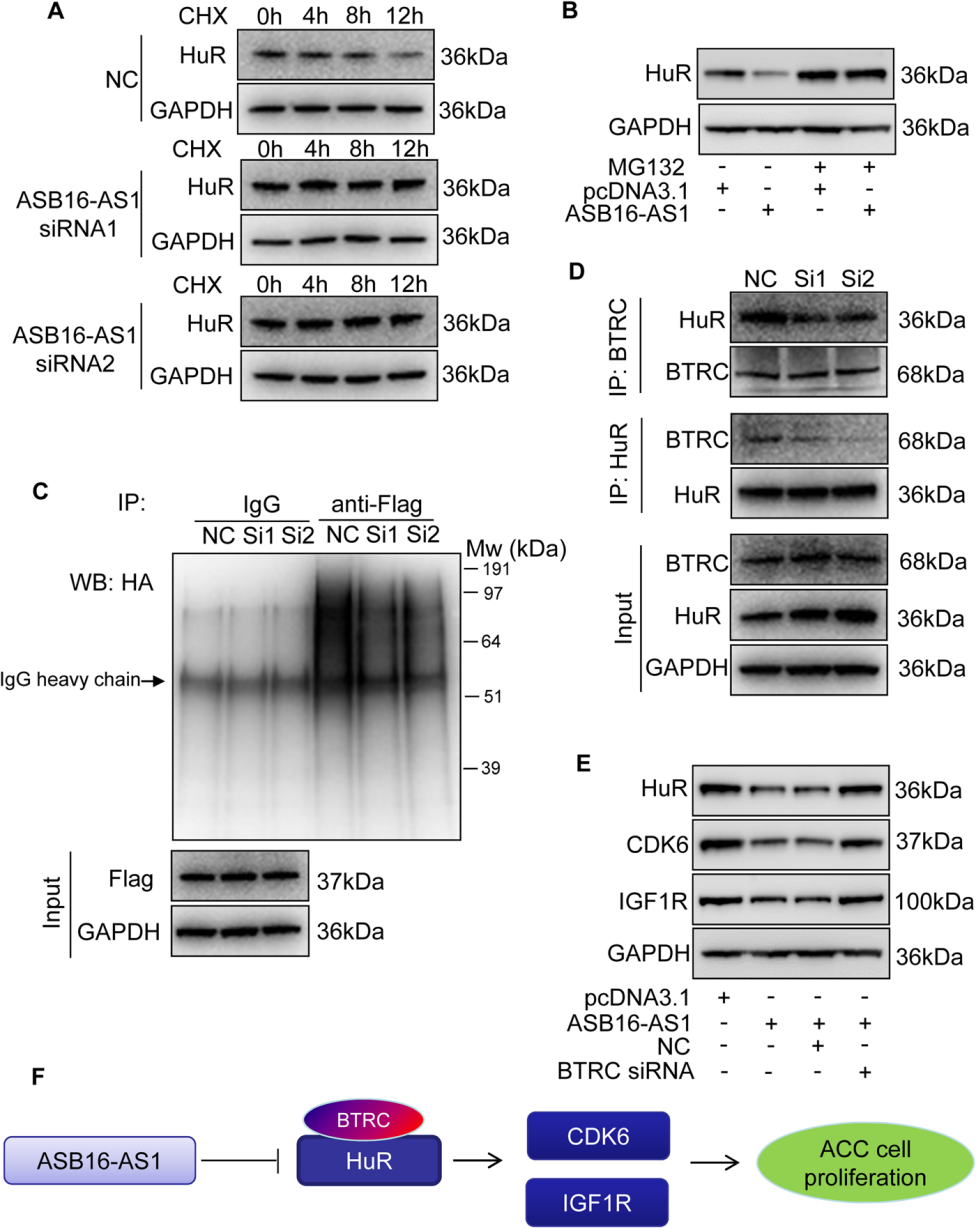
ASB16-AS1 post-translationally represses HuR expression through BTRC-mediated ubiquitination. **a** Inhibition of ASB16-AS1 expression attenuates HuR protein degradation. SW-13 cells were transfected with ASB16-AS1 siRNA1 or siRNA2 or its negative control (NC) and then treated with 50 μg/ml cycloheximide (CHX) for the indicated period of time. HuR protein levels were detected by western blot. **b** ASB16-AS1 promotes HuR degradation via proteasomal degradation. SW-13 cells were transfected with a plasmid encoding ASB16-AS1 or pcDNA3.1 control and cells were treated with 20 μM MG132 for 24 h. HuR protein levels were detected by western blot. **c** ASB16-AS1 promotes HuR protein ubiquitination. SW-13 cells were co-transfected with HA-Ub, Flag-HuR, ASB16-AS1 siRNA1 (Si1), or ASB16-AS1 siRNA2 (Si2) or its negative control (NC), the cells were then treated with 20 μM MG132 for 24 h. The cells were then lysed for immunoprecipitation. Immunoprecipitation was performed using anti-Flag antibody. IgG served as negative control. Western blot was used to detect ubiquitinated HuR protein using anti-HA antibody. **d** Inhibition of ASB16-AS1 expression attenuates the interaction between HuR and the ubiquitin E3 ligase BTRC. SW-13 cells were transfected with ASB16-AS1 siRNA1 (Si1) or ASB16-AS1 siRNA2 (Si2), immunoprecipitation was performed using either anti-HuR or anti-BTRC antibody, western blot was performed to detect the association between HuR and BTRC protein. **e** Knockdown of BTRC abolished down-regulation of HuR, CDK6, IGF1R upon enhanced expression of ASB16-AS1. Adrenocortical carcinoma SW-13 cells were transfected with plasmid encoding ASB16-AS1 or in combination with siRNA targeting BTRC, 48 h post-transfection the expression of HuR, CDK6, and IGF1R were detected by western blot. **f** Schematic of ASB16-AS1 regulating adrenocortical carcinoma cell proliferation. Three independent experiments were performed.

We then elucidate how ASB16-AS1 modulates HuR protein expression. Protein ubiquitination modification is an important post-translational method to modulate protein levels. We employed a protein synthesis inhibitor cycloheximide (CHX) to treat adrenocortical carcinoma cells and the results showed that HuR protein degradation is significantly inhibited upon ASB16-AS1 knockdown (Fig. 7a). This indicates that ASB16-AS1 post-translationally regulates HuR protein expression. To figure out whether ASB16-AS1 regulates HuR protein levels through ubiquitination, we treated adrenocortical carcinoma cells with a specific proteasome inhibitor MG132, and the results showed that MG132 treatment abolished the down-regulation of HuR protein expression upon overexpression of ASB16-AS1 (Fig. 7b). This result indicates that HuR is a proteasome substrate and these data showed that ASB16-AS1 post-translationally regulate HuR protein expression by modulating HuR protein degradation.

To elucidate the mechanism how ASB16-AS1 regulate HuR protein degradation, we co-transfected adrenocortical carcinoma cells with HA-tagged ubiquitin and a plasmid expressing Flag-tagged HuR. The results demonstrate that HuR ubiquitination level decreased upon knockdown of endogenous ASB16-AS1 (Fig. 7c). Studies have found that HuR is the substrate protein of E3 ubiquitin ligase BTRC and HuR is subjected for ubiquitination and degradation^14,16^. To study whether ASB16-AS1 post-translationally regulates HuR expression through BTRC, we performed co-IP experiments to detect whether ASB16-AS could affect the association between BTRC and HuR. The results turned out that inhibition of ASB16-AS1 reduces the interaction between BTRC and HuR. We obtained the same results using either antibody of BTRC or HuR to perform immunoprecipitaton (Fig. 7d). In summary, these data demonstrate that ASB16-AS1 post-translationally inhibits HuR protein expression through E3 ligase BTRC-mediated ubiquitination and degradation. ASB16-AS1 promoted the interaction between HuR and BTRC, suggesting BTRC is necessary for the effect of ASB16-AS1 on HuR, CDK6, and IGF1R. We designed siRNA targeting BTRC (Supplementary Fig. S3e, f) and knocked down BTRC expression in ASB16-AS1-overexpressed cells to test if BTRC knock-down could reverse the effect of AB16-AS1 on HuR, CDK6, and IGF1R levels. The results turned out that knock-down of BTRC abolished the down-regulation of HuR, CDK6, and IGF1R expression upon enhanced expression of ASB16-AS1 in adrenocortical carcinoma cells (Fig. 7e).

## Discussion

In this study, we elucidated the molecular mechanism of ASB16-AS1 inhibiting adrenocortical carcinoma cell proliferation and tumor growth. By RNA pull-down following mass spectrometry, we found that ASB16-AS1 associates with RNA-binding protein HuR which enhances the expression of CDK6 and IGF1R mRNAs. Knockdown of ASB16-AS1 post-translationally promote the expression of HuR protein. In addition, we found that inhibition of ASB16-AS1 inhibits HuR protein degradation by reducing the interaction between E3 ligase BTRC and HuR. Our findings provide a novel target to treat adrenocortical carcinoma.

Studies have found that ASB16-AS1 promotes cell proliferation in glioma, hepatocellular carcinoma, cervical cancer, non-small cell lung cancer and in osteosarcoma^17–21^. However, the biological function and molecular mechanism of ASB16-AS1 in adrenocortical carcinoma remains unknown. In our study, we found ASB16-AS1 is down-regulated in adrenocortical carcinoma and inhibits adrenocortical carcinoma cell proliferation. From GEPIA database, ASB16-AS1 is up-regulated in hepatocellular carcinoma, whereas it is down-regulated in adrenocortical carcinoma (Fig. 1a). Our transcriptome RNA-sequencing results showed that inhibition of ASB16-AS1 promotes the expression of genes involved in cell cycle progression and cell proliferation in adrenocortical carcinoma cells. This indicates that ASB16-AS1 may exert different functions in different cancers, which are dependent on cell type and the tumor cellular context.

Studies have found that dysregulation of lncRNAs plays a vital role in tumor initiation and progression, however, the molecular mechanism that a lncRNA exerts its function is complex and remains to be challenge to clarify^28^. Many studies have found that lncRNA functions as competing endogenous RNA that regulates the expression or activities of miRNAs and subsequently regulate miRNA target expression^29^. In fact, lncRNA can function in *cis* or in *trans*. Studies have found that lncRNAs can interact with proteins, regulate the expression of the protein it interacts^6,30^. Study found that lncRNA-OCC1 can interact with HuR and inhibit HuR protein expression post-translationally^14^. In our study, we used RNA pull-down following mass spectrometry and found that ASB16-AS1 associates with RNA-binding protein HuR and regulate the expression of HuR post-translationally.

The RNA-binding protein HuR can interact with various species of RNAs, including coding and non-coding RNA transcripts. HuR can be post-translationally modified, it can be phosphorylated, methylated, or ubiquitinated^10^. Ubiquitination is an important way of post-translational modification that participates in the regulation of various cellular processes, including cell survival and cell differentiation. The ubiquitin proteasome system is delicately regulated and it selectively markers protein for degradation in the cell. Ubiquitination is orchestrated by ubiquitin-activating enzymes (E1s), ubiquitin-conjugating enzymes (E2s), and ubiquitin ligases (E3s). Dysregulation of ubiquitination affects tumor cell cycle regulation, gene expression, and tumor progression^15,31–33^. Recently, studies have found that lncRNAs can mediate ubiquitination pathway and regulate the expression of target proteins. LncRNA GBCDRlnc1 directly interacts with phosphoglycerate kinase 1 (PGK1) and increasing its protein level by inhibiting PGK1 ubiquitination in gallbladder cancer cells^34^. LINC00673 directly interacts with tyrosine phosphatase non-receptor type 11 (PTPN11) and functions as tumor suppressor in pancreatic cancer. It enhances the interaction between PTPN11 and E3 ligase PRPF19 and promoting PTPN11 degradation by ubiquitination^35^. LINC01638 interacts with c-Myc and inhibit E3 ubiquitin ligase adapter speckle-type POZ (SPOP)-mediated protein degradation of c-Myc in breast cancer^36^. LINC02023 binds with PTEN and prevents degradation which is mediated by E3 ubiquitin ligase WWP2 in colorectal cancer^37^. In this study, we found that ASB16-AS1 post-translationally regulates the protein levels of HuR by enhancing E3 ligase BTRC binding with HuR and subsequently degradation of HuR protein.

CDK6 and IGF1R are important regulators of cancer progression. The insulin-like growth factor-1 receptor is a potent pro-survival tyrosine kinase-containing receptor and is critical for cancer cell survival and participates in tumorigenesis. IGF1R has become a therapeutic target for many caners^24,25^. Cell cycle progression is controlled by cyclin-dependent kinases (CDKs). CDK6 is an important cell cycle regulator controlling cell cycle transition from G0/G1 to S-phase^26,38^. Study found that Lnc-UCID promotes CDK6 expression by preventing the interaction of DHX9 with CDK6, and subsequently promoted G1/S transition and cell proliferation in hepatocellular carcinoma^39^. In this study, we found that lncRNA ASB16-AS1 inhibits adrenocortical carcinoma cell cycle progression and cell proliferation by inhibiting CDK6 and IGFR expressions, which is mediated by RNA-binding protein HuR. Recently, a study found that combined treatment with IGF1R and CDK4/6 inhibitors showed enhanced suppression of cell cycle in Ewing sarcoma^40^. Our study found that ASB16-AS1 inhibits IGF1R and CDK6 in adrenocortical carcinoma, modulating of their levels may be developed to treat this type of carcinoma.

HuR is a RNA-binding protein that interacts and stabilizes target RNAs. It can bind mRNAs and lncRNAs. HuR is abundant in different cancer cells. Study found that HuR interacts with 5′UTR of IGF1R mRNA and subsequently repressed translation initiation through the IGF1R 5′ UTR^41^. While another study found that HuR bind with IGF1R mRNA and increase the stability of mRNA. The sequestration of HuR by pVHL decreases the stability of IGF1R mRNA in human clear cell renal carcinoma^42^. Our data demonstrate that HuR interacts with IGF1R mRNA, knockdown of HuR reduces IGF1R mRNA and subsequently protein expression.

In summary, our study found that ASB16-AS1 is down-regulated in adrenocortical carcinoma and it inhibits adrenocortical carcinoma cell cycle progression and proliferation in vitro and in vivo animal experiments. ASB16-AS1 interacts with HuR and promotes the binding of BTRC with HuR protein and finally promotes the ubiquitination and degradation of HuR. Our study revealed a novel signaling pathway that controlling adrenocortical carcinoma cell growth, and ASB16-AS1 may become a new therapeutic target to treating this cancer.

## Supplementary information

Supplementary figure S1

Supplementary figure S2

Supplementary figure S3

Supplementary figure legends

Supplementary Table 1

Supplementary Table 2

Supplementary Table 3-6
