# Supplementary material for: Long noncoding RNA ASB16-AS1 inhibits adrenocortical carcinoma cell growth by promoting ubiquitination of RNA-binding protein HuR
Source: Cell Death Dis. 2020 Nov 20;11(11):995. doi: 10.1038/s41419-020-03205-2 (PMC7679391; doi:10.1038/s41419-020-03205-2)
Supplement: Supplementary file 4 — Supplementary figure legends [file 41419_2020_3205_MOESM4_ESM.docx]

**Supplementary figure legends**

**Fig. S1 LncRNA ASB16-AS1 inhibits adrenocortical carcinoma cell proliferation. a** The relative expression of ASB16-AS1 was detected by qRT-PCR after transfection of a plasmid expressing ASB16-AS1 in adrenocortical carcinoma cells SW-13 and H295R respectively. pcDNA3.1 served as control in the experiment. **P* < 0.05 versus pcDNA3.1 group. **b** Adrenocortical carcinoma cell proliferation is inhibited upon enhanced expression of ASB16-AS1 as revealed by CCK-8 assay. SW-13 or H295R cells were transfected with a plasmid expressing ASB16-AS1, cell proliferation was analysed by CCK-8 kit at the indicated time point post transfection. **P* < 0.05 versus pcNDA3.1 group. **c** ASB16-AS1 inhibits adrenocortical carcinoma cell proliferation determined by EdU assays. Plasmids expressing ASB16-AS1 were transfected into SW-13 or H295R cells and cell proliferation was determined by EdU assay. Scale bar equals 100 μm. **P* < 0.05 versus pcNDA3.1 group. **d, e** ASB16-AS1 repress cell cycle progression in adrenocortical carcinoma cells. Adrenocortical carcinoma cell SW-13 or H295R cells were transfected with a plasmid expressing ASB16-AS1 and cell cycle was analysed by FACS assay. pcDNA3.1 served as control. **P* < 0.05 versus pcDNA3.1 group. The experiments were performed in triplicate. The data are represented as mean ± SEM from three independent experiments.

**Fig. S2 ASB16-AS1 distribution in adrenocortical carcinoma cells and construction of adrenocortical carcinoma cells stably expressing ASB16-AS1. a, b** ASB16-AS1 distributed in both nucleus and cytoplasm in adrenocortical carcinoma cells. SW-13 or H295R cells were fractionated to isolated RNA from nucleus and cytoplasm. The relative ASB16-AS1 level was detected by qRT-PCR. U6 and GAPDH served as nucleus and cytoplasm control, respectively. **c** Construction of adrenocortical carcinoma cells stably expression ASB16-AS1. SW-13 cells were infected with a lentivirus encoding ASB16-AS1 or a control lentivirus, the cells were then screened by puromycin. The expression of ASB16-AS1 was detected by qRT-PCR. **P* < 0.05 versus control. The experiments were performed in triplicate. The data are represented as mean ± SEM from three independent experiments.

**Fig. S3 Enhanced expression of ASB16-AS1 down-regulates the protein levels of CDK6 and IGF1R and knockdown of BTRC in adrenocortical carcinoma cells. a**, **b** Enhanced expression of ASB16-AS1 reduces mRNA levels of CDK6 and IGF1R. SW-13 or H295R cells were transfected with a plasmid encoding ASB16-AS1, the expression of CDK6 and IGF1R mRNAs were detected by qRT-PCR forty-eight hours post transfection. pcDNA3.1 served as control. **P* < 0.05 versus pcDNA3.1 group. **c**, **d** ASB16-AS1 expression plasmids were transfected into SW-13 or H295R cells, the protein levels of CDK6 and IGF1R were analysed by western blot. pcDNA3.1 served as control. **e**, **f** BTRC was efficiently knocked down in adrenocortical carcinoma cells. siRNA targeting BTRC was transfected into adrenocortical carcinoma cell SW-13 cells, the mRNA and protein levels were detected by qRT-PCR and western blot 72 hours post transfection. **P* < 0.05 versus NC group. The experiments were performed in triplicate. The data are represented as mean ± SEM from three independent experiments.
