# Supplementary material for: Long noncoding RNA ASB16-AS1 inhibits adrenocortical carcinoma cell growth by promoting ubiquitination of RNA-binding protein HuR
Source: Cell Death Dis. 2020 Nov 20;11(11):995. doi: 10.1038/s41419-020-03205-2 (PMC7679391; doi:10.1038/s41419-020-03205-2)
Supplement: Supplementary file 7 — Supplementary Table 3-6 [file 41419_2020_3205_MOESM7_ESM.doc]

**Supplementary Table 3. Primers used for qRT-PCR**

| Gene | Forward primer | Reverse primer |
| --- | --- | --- |
| ASB16-AS1 | GACAACAGAATTGGAAGGTCC | CTGTCTGAGGCAGTGAGTAC |
| CDK6 | TGAGGCACCTGGAGACCTTCG | GGCACTCCAGGCTCTGGAAC |
| IGF1R | GTACAACTACCGCTGCTGGACC | TACACAGGCCGTGTCGTTGTC |
| GAPDH | AACGTGTCAGTGGTGGACCTG | GAGACCACCTGGTGCTCAGTG |
| HuR | AAGGACGTAGAAGACATGTTC | GTTTGTCAAACCGGATAAAC |
| BTRC | CAAGCACTGCTATGAAGACTGAG | ATCTGACTCTGACCACTGCTC |

**Supplementary Table 4. siRNA sequence used to knock-down the expression of ASB16-AS1, HuR and BTRC in adrenocortical carcinoma cells**

| Name | Forward sequence | Reverse sequence |
| --- | --- | --- |
| ASB16-AS1 siRNA-1 | AGGUCUGGUUCUGAAUCAUUC | GAAUGAUUCAGAACCAGACCU |
| ASB16-AS1 siRNA-2 | ACCCACUUCUGUGUACGUUAU | AUAACGUACACAGAAGUGGGU |
| HuR siRNA | CGUAAGUUAUUUCCUUUAAdTdT | UUAAAGGAAAUAACUUACGdTdT |
| BTRC siRNA | GGUCAGAGUGUGGGAUGUAAA | UUUACAUCCCACACUCUGACC |

**Supplementary Table 5. The sequences of ASB16-AS1 fragment F1 and F2 used in RNA pull-down experiments (The red sequence is potential HuR binding sites)**

**ASB16-AS1 Fragment 1 (F1)**

GTGGCTTCGCGACTGCGGAAGGTTGCCCGGGCTGGCGCCGCTGGGCAGAGCCGGTGCGACGTTTCCCTTTCCAGCTTTGTTCTTCCGGCCCTGCTGTCTGCCTCCCCGGCTGATTGGATTCGTTACTGCTTTGCAACGCGCACCTAACTCCAGGCTGGAATGCAATAGCGTGGTCTTGGCTCACTGCAACCTCCGCCTCCCGGGTTCAAGCAATTCTCCTGCCTCAGCCTCCTAAGTAGCTGGGACTACAGCTTGAGGTCCTACCTGGTGTCCTCCATGAGAATCATACAAGCCCCGCTACAGACAACAGAATTGGAAGGTCCTGAGAGCTACCAGAAGCATCTTCAGTTTTCATATGAAAAACTTAAGCCCAGGAGTGATTTGTGCAGCCAAGGACACACAGCAAGTTAGTGGCTGCACCAGAAATAGTACTCACTGCCTCAGACAGTCCCTCCCACAGTCTACTCTGTCTCCACCCCAAGGTCTG

**ASB16-AS1 Fragment 2 (F2)**

GTTCTGAATCATTCAGTTGTCAGTTGTCTCCTTTTTTTTTTTTTTTTTTGGAGACAGGGTCTCTCTCTGTGGCCCAGGCTGAAGTGCAGTGGTGTGATTACAGCTCATGTAGCCTGGACCTCCCAAGCTCAAGCAATCCTCCTGCCTCTGCCTCCCAAGTAGCTGGGACCACAGGTGGGCAGCACCACACCTGGCTAATTTTTTAAATTTTTTTTTATAGAGACAGGGTCTTACCATTTGCCCCAGCTGGTCTCAAACCCCTGGGCTCAAGCAAGCCTCCTGCCTTGGCCTCCCAAAGTGCTGGGATTACAGGTGTGAGCCACCACACCCGGCCTTATCTCCTAACATTTTGATAGCACAAAATTTTGAAAACCCACTTCTGTGTACGTTATGAAGCATAGAAAATTAAACAGTATGCACCAAAAAAAAATAAAAA

**Supplementary Table 6. The full name of different tumor names used in Fig. 1a.**

| **Abbreviations** | **Full name** |
| --- | --- |
| ACC | adrenocortical carcinoma |
| BLCA | bladder urothelial carcinoma |
| BRCA | breast invasive carcinoma |
| CESC | cervical squamous cell carcinoma and endocervical adenocarcinoma |
| CHOL | cholangio carcinoma |
| COAD | colon adenocarcinoma |
| DLBC | lymphoid neoplasm diffuse large B-cell Lymphoma |
| ESCA | esophageal carcinoma |
| GBM | glioblastoma multiforme |
| HNSC | head and neck squamous cell carcinoma |
| KICH | kidney chromophobe |
| KIRC | kidney renal clear cell carcinoma |
| KIRP | kidney renal papillary cell carcinoma |
| LAML | acute myeloid Leukemia |
| LGG | brain lower grade glioma |
| LIHC | liver hepatocellular carcinoma |
| LUAD | lung adenocarcinoma |
| LUSC | lung squamous cell carcinoma |
| MESO | mesothelioma |
| OV | ovarian serous cystadenocarcinoma |
| PAAD | pancreatic adenocarcinoma |
| PCPG | pheochromocytoma and paraganglioma |
| PRAD | prostate adenocarcinoma |
| READ | rectum adenocarcinoma |
| SARC | sarcoma |
| SKCM | skin cutaneous melanoma |
| STAD | stomach adenocarcinoma |
| TGCT | testicular germ cell tumors |
| THCA | thyroid carcinoma |
| THYM | thymoma |
| UCEC | uterine corpus endometrial carcinoma |
| UCS | uterine carcinosarcoma |
